# Hypothalamic effective connectivity at rest is associated with body weight and energy homeostasis

**DOI:** 10.1162/netn_a_00266

**Published:** 2022-10-01

**Authors:** Katharina Voigt, Zane B. Andrews, Ian H. Harding, Adeel Razi, Antonio Verdejo-García

**Affiliations:** School of Psychological Sciences and Turner Institute for Brain and Mental Health, Monash University, Victoria, Australia; Biomedicine Discovery Institute and Department of Physiology, Monash University, Victoria, Australia; Department of Neuroscience, Central Clinical School, Monash University, Melbourne, Australia; The Wellcome Centre for Human Neuroimaging, University College London, London, UK

**Keywords:** Resting-state fMRI, Spectral dynamic causal modelling, Effective connectivity, Energy homeostasis, Obesity

## Abstract

Hunger and satiety drive eating behaviours via changes in brain function. The hypothalamus is a central component of the brain networks that regulate food intake. Animal research parsed the roles of the lateral hypothalamus (LH) and medial hypothalamus (MH) in hunger and satiety, respectively. Here, we examined how hunger and satiety change information flow between human LH and MH brain networks, and how these interactions are influenced by body mass index (BMI). Forty participants (16 overweight/obese) underwent two resting-state functional MRI scans while being fasted and sated. The excitatory/inhibitory influence of information flow between the MH and LH was modelled using spectral dynamic causal modelling. Our results revealed two core networks interacting across homeostatic state and weight: subcortical bidirectional connections between the LH, MH and the substantia nigra pars compacta (prSN), and cortical top-down inhibition from fronto-parietal and temporal areas. During fasting, we found higher inhibition between the LH and prSN, whereas the prSN received greater top-down inhibition from across the cortex. Individuals with higher BMI showed that these network dynamics occur irrespective of homeostatic state. Our findings reveal fasting affects brain dynamics over a distributed hypothalamic-midbrain-cortical network. This network is less sensitive to state-related fluctuations among people with obesity.

## Author Summary

The hypothalamus is a central component of the brain networks regulating food intake. Animal research subdivided the hypothalamus anatomically and functionally into lateral hypothalamus (LH) and medial hypothalamus (MH). This is the first study showing how the LH and MH causally interact with other neural regions and how their dynamics change with weight and homeostasis in humans. Adopting state-of-the-art spectral dynamic causal modelling of resting-state fMRI data, we provide new insights into how homeostasis affect hypothalamic circuit dynamics, which involve a distributed network of midbrain and cortical areas with a key role of the substantia nigra. We identified unique aspects of network organisation associated with obesity involving reciprocal connections between the LH and MH, and input from the substantia nigra to the MH.

## INTRODUCTION

The hypothalamus accounts for only approximately 3% of total human brain tissue, but is one of the most vital structures regulating a plethora of bodily functions essential for survival (Stuber & Wise, 2016). This small subcortical region regulates our response to stress, arousal, reward processing, body temperature, fertility and sexual behaviour, motivation, and food intake (Gabriela Pop et al., 2018). Early preclinical lesion studies subdivided the hypothalamus anatomically and functionally into lateral hypothalamus (LH) and medial hypothalamus (MH), leading to the concept of a “dual centre model” (Anand & Brobeck, 1951; Brobeck et al., 1943; Elmquist et al., 1999; Hetherington & Ranson, 1983). Lesions to the MH resulted in increased appetite, food intake and weight gain, marking the MH as “satiety centre.” Lesions to the LH in turn induced abnormal decreases in appetite and food intake, labelling the LH as “hunger centre.” More recent studies show that neurons in the LH regulate food consumption and appetitive motivation with extensive reciprocal connections to the dopaminergic midbrain governing reward processing in support of goal-directed food seeking (Jennings et al., 2013, 2015; Nieh et al., 2016; Rossi & Stuber, 2018). The LH is a large single region with numerous heterogeneous neuronal populations, whereas the MH can be further subdivided into many important nuclei involved in the regulation of food intake, blood glucose, and weight control. This includes the arcuate nucleus, the ventromedial hypothalamic nucleus, the dorsomedial hypothalamic nucleus, and the paraventricular nucleus. Both the LH and MH nuclei function in a metabolic state-dependent manner and can be reshaped by obesity and energy homeostasis (Chen et al., 2017; Rossi et al., 2019). Moreover, these hypothalamic areas are heavily integrated into intra- and interhypothalamic neural circuits and networks, with the majority of LH connectivity coming from outside the LH (Burdakov & Karnani, 2020).

Although animal research has greatly contributed to understanding how hypothalamic neural circuits integrate peripheral and central signals to control food intake, the connectivity in humans to and from the LH and MH nuclei remains poorly understood. Energy homeostasis relies on the coordinated and dynamic interactions of the hypothalamus both to (bottom-up) and from (top-down) a broad set of cortical and subcortical brain regions (Rossi & Stuber, 2018). A precise description of how the LH and MH network functions in response to changes in homeostatic state in humans is thus required to bridge the gap between animal and human research, and to provide a critical step towards defining the neural underpinnings of maladaptive eating patterns leading to obesity in humans. An examination of the LH and MH networks is further supported by the well-known psychological comorbidities associated with metabolic diseases such as anorexia, obesity, and diabetes (Florent et al., 2020; Penninx & Lange, 2018).

Research has begun to establish the links between the hypothalamic network and obesity using functional magnetic resonance imaging (fMRI). One study by Kullmann and colleagues (2014) described differences in the hypothalamic network between people with excess weight and those with healthy weight. Functional connectivity analyses revealed the LH was more heavily connected to the dorsal striatum, anterior cingulum, and frontal operculum, and the MH was more connected to the medial orbitofrontal cortex and nucleus accumbens (replicated recently by Zhang et al., 2018). Further, in participants with excess weight, the functional connectivity of the MH, but not the LH, was increased with the nucleus accumbens and medial prefrontal cortex. These results highlight the existence of two distinct circuitries originating from the MH and LH that are modulated by obesity. However, these studies do not reveal the functional interactions (e.g., inhibition or excitation) nor do they differentiate between bottom-up and top-down effects within the network. Further, given the metabolic state-dependency of the LH and MH circuitries (Chen et al., 2017; Rossi et al., 2019), it is also critical to investigate this network as a function of more dynamic state-dependent changes in energy homeostasis, such as in states of fasting versus satiety.

The current study examines the directionality (bottom-up vs. top-down) and valence (inhibition vs. excitation) of connections of the LH and MH with key cortical and subcortical brain regions. These network dynamics are examined in participants varying in weight (healthy vs. excess weight) in a fasted or sated state. We capitalise on recent advances in modelling the interactions within a brain network based on the low-frequency endogenous fluctuations in resting-state functional magnetic resonance imaging (rsfMRI) data using spectral dynamic causal modelling (spDCM; Friston et al., 2014; Razi et al., 2015; Park et al., 2018) and state of the art anatomical labelling (Rolls et al., 2020). In contrast to conventional functional connectivity analyses (e.g., Kullmann et al., 2014; Zhang et al., 2018), spDCM predicts directional communications among distributed brain regions (i.e., effective connectivity; Friston, Harrison, & Penny, 2003). We hypothesise that the LH and MH would show distinct effective connectivity. Specifically, based on reviewed previous functional connectivity studies distinguishing between MH and LH (Kullmann et al., 2014; Zhang et al., 2018), we predicted that the (a) LH might be more heavily interconnected than the MH with the dorsal striatum, anterior cingulum, and frontal operculum, and (b) that these connections from and to the MH, but not LH, are affected by BMI. Given that there are no previous fMRI studies investigating the network dynamics of the LH and MH across homeostatic state, how hunger and satiety affect the hypothalamic network dynamics was explorative.

## METHODS

### Participants

Forty participants were recruited via flyers and social media advertisements. Participants were required to be 18–55 years old, right-handed, and have a body mass index (BMI) between 18 and 30 kg/m^2^. Screening criteria excluded people with a history of hypertension or diabetes, neurological or psychiatric illness, or who had recently taken psychoactive medications. Additionally, participants could not be subject to MRI contradiction, such as metal implants or pregnancy. The number of participants was chosen based on a sample size estimation study revealing that 20 participants provided for reliable DCM predictions (Goulden et al., 2012). In agreement, recent research showed robust model predictions using similar sample sizes when applying spDCM to rsfMRI data (Park et al., 2018; Preller et al., 2019; Voigt et al., 2020). Out of the 40 participants, two were excluded from analysis as they did not complete both fasted and sated rsfMRI scans. In total, data from 38 participants were included into the analyses (Table 1 for participants’ demographics). From these participants, 22 had healthy weight (18.56–24.27 kg/m^2^), 4 were overweight (25.12–28.82 kg/m^2^), and 12 were obese (30.84–55.55 kg/m^2^). All participants gave written consent before participating and were reimbursed with $100 gift card vouchers. The Monash University Human Research Ethics Committee approved the study (2019-5979-30222) following the Declaration of Helsinki.

**Table 1.** Participants’ demographics

Characteristic	*M*	*SD*	Range
Age	27.40	8.10	18–48
Female/male	25/13		
Obese and overweight/healthy weight^a^	16/22		
BMI^b^	26.44	7.16	18.56–55.56
Hip-waist ratio^c^	0.92	0.10	0.79–1.25
Blood glucose^d^	4.94	0.79	3.8–8.4
Hunger fasted^e^	4.41	1.44	1.18–7
Hunger sated^e^	3.22	1.48	1.45–6.21

*Note*. *M* = mean; *SD* = standard deviation; BMI = body mass index.

^a^ Healthy weight = BMI between 18–25 kg/m^2^; Obese = BMI from 30 kg/m^2^.

^b^ BMI = weight (kg)/height (m^2^).

^c^ Waist circumference divided by hip circumference.

^d^ Blood glucose levels were assessed via finger prick test conducted at the start of the fasted condition.

^e^ Hunger levels were based on self-report via a 1 (not at all) to 7 (very much) Likert scale.

### Experimental Procedure

Participants completed two resting-state fMRI scans, one after an overnight fast (fasted condition) and one after a standard breakfast (sated condition). In both conditions, participants were instructed to have a standard meal (700–1,000 kJ) between 7.30 pm and 8.30 pm on the night prior to their scan and subsequently to refrain from eating or drinking (except for water) until their morning scan. Fasting blood glucose levels were measured via a standard finger prick test. For the sated condition, participants received a breakfast (293 kcal) 1 hour prior to their scan. Subjective self-reports of hunger (1 = not hungry at all; 7 = very hungry) revealed a significant difference in the perception of hunger during the fasted (*M* = 4.42; *SD* = 1.44) and sated (*M* = 3.22; *SD* = 1.48) condition (*t*(36) = 4.72, *p* < 0.001). There was no interaction between subjective reported hunger and BMI, but there was between BMI and fasting blood glucose level (see Supporting Information Figure S1 and Figure S2). All scans were scheduled in the morning between 9 am and 10 am. On average there were 5.82 days (*SD* = 3.73 days) between the two scanning sessions. The order of fasted and sated scans was counterbalanced across participants.

### Resting-State fMRI Data Acquisition

Resting-state fMRI data were acquired using a 3-Tesla Siemens Skyra MRI scanner equipped with a 32-channel head coil at the Monash Biomedical Imaging Research Centre (Melbourne, Victoria, Australia). During a total acquisition time of 7.8 minutes, 600 volumes were acquired for each participant and homeostatic condition using a multiband gradient echo pulse sequence (45 axial slices; time of repetition, TR = 780 ms; echo time, TE = 21 ms, resolution 3 × 3 × 3 mm). In order to obtain structural brain information for each participant, a high-resolution T1-weighted magnetisation-prepared rapid gradient echo covering the whole brain was measured (repetition time = 2,300 ms; echo time = 2.07 ms; flip angle = 9°; 192 slices; field of view = 256 × 256 mm, voxel resolution = 1 mm isotropic). Participants were instructed to rest while fixating on a central black crosshair (i.e., eyes-open resting-state protocol).

### Resting-State fMRI Data Analyses

Functional images were preprocessed using SPM12 (revision 12.2, www.fil.ion.ucl.ac.uk). The preprocessing steps consisted of spatial realignment, tissue segmentation, and spatial normalisation to the standard EPI template of the Montreal Neurological Institute (MNI), and spatial smoothing using a Gaussian kernel of 6-mm FWHM. None of the participants exceeded excessive head motion of larger than 3 mm. For the seed-based functional connectivity analyses, we applied an additional temporal band-pass filter (0.01–0.08 Hz) to remove low-frequency drifts and high-frequency physiological noise as well as linear detrending the data. Nuisance covariate regression was performed to remove signal variance of nonneuronal origin using time series extracted from the white matter, and independently from the cerebrospinal fluid, in addition to the six parameters to define the magnitude of frame-by-frame head motion (3 × translation; 3 × rotation).

### Statistical fMRI Analyses

**Figure 1.**
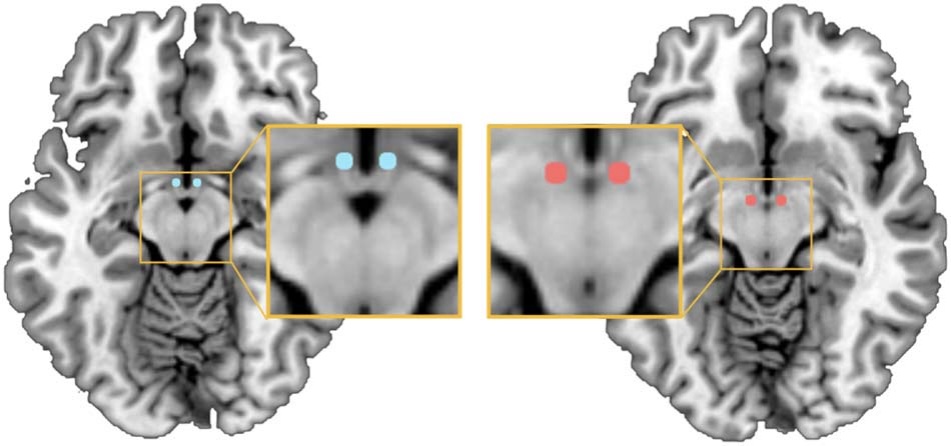
Seed regions of the medial hypothalamus (MH; MNI coordinates *x* = ±4, *y* = −2, *z* = −12; left depicted in blue), and lateral hypothalamus (LH; MNI coordinates *x* = ±6, *y* = −9, −10; right depicted in red) (based on Baroncini et al., 2012) used to obtain the hypothalamic network for subsequent spDCM analyses.

We first conducted an initial seed-based functional connectivity analysis (using the bilateral MH and LH as seeds; Figure 1). This analysis was used to obtain the brain areas that are associated with the MH and LH at rest (i.e., the hypothalamic functional resting-state network). Next, we conducted a spDCM analyses to investigate the causal interactions between these areas and how they differ as a function of homeostatic state (fasted vs. sated), BMI, and the interaction between homeostatic state and BMI. The details of these two analyses are outlined next.

### Seed-Based Functional Connectivity Analysis

Functional connectivity maps of the hypothalamic functional resting-state network were obtained using an initial seed-based functional connectivity analyses across all subjects based on the data obtained during the fasted condition (Kullmann et al., 2014). We defined two ROIs according to Baroncini et al. (2012): the bilateral LH; MNI coordinates *x* = ±6, *y* = −9, *z* = −10) and bilateral MH; MNI coordinates *x* = ±4, *y* = −2, *z* = −12) using 2-mm-radius spheres (Figure 1). To minimise overlap between the two ROIs, we chose the peak voxel of the LH to be in the posterior part of the LH according to Baroncini et al. (2012). The seeds were, as such, spatially separated by >6 mm (i.e., >1 mm after smoothing).

In order to define the general hypothalamus network that was associated with either of the LH or MH across subjects, we extracted the average time series from LH and MH combined. This time series was then correlated with the time series of activity within each of voxel across the rest of the brain. The resulting functional connectivity maps were transferred to *z*-scores using Fisher’s transformation and analysed using a one-sample’s *t* test in SPM12 (Wellcome Department of Cognitive Neurology, London, UK). Brain voxels with a threshold of *p* < 0.05, family-wise error (FWE) corrected for multiple comparisons on the voxel-level were considered significant. Anatomical regions were labelled using the recent update of the automatic anatomical labelling atlas AAL3 (Rolls et al., 2020). This updated atlas includes brain areas that have not generally been defined in other atlases, such as subdivisions of the thalamus or the substantia nigra. Previous studies investigating the functional connectivity of the MH and LH (Kullmann et al., 2014; Zhang et al., 2018) have not used such precise anatomical labelling.

### Spectral Dynamic Causal Modelling

The spDCM analyses were performed using the functions of DCM12 (revision 7196) implemented in SPM12 (version 7487) in MATLAB 2018b. In order to address our main hypotheses, we focused on spDCM analyses that assessed four questions: (1) effective connectivity of the hypothalamic network in the fasted and sated states independently; (2) changes in hypothalamic effective connectivity between the fasted versus sated state, independent of BMI (main effect of fasting); (3) changes in hypothalamic effective connectivity modulated by BMI, independent of energy state (main effect of BMI); and (4) changes in fasting-related effective connectivity of the hypothalamus modulated by BMI (fasting-by-BMI interaction). Question (1) will be assessed via the first-level spDCM analysis and Question (2)–(4) will be assessed via the second-level spDCM analyses. The first level of the spDCM is an intercept model, which estimates the direction and valence (i.e., inhibition/excitation) regardless of any behavioural variable (e.g., BMI, homeostasis). As such, it estimates the neuronal network dynamics of our chosen network (as established via an initial functional connectivity analyses) during the resting-state fMRI whilst individuals where fasted and sated. On the next level, we estimated additional spDCM to assess the effects of BMI and homeostasis and their interaction on the neuronal network dynamics.

In DCM, each connection has a prior distribution, which assumes them to have a normal (Gaussian) distribution (the so-called Laplace approximation). The priors on connectivity parameters are given in Table 1 of Friston et al. (2014). Each extrinsic (between-region) connection has a small but positive prior mean value (i.e., excitatory connectivity) but with a variance that allows it to take on a posterior connectivity which can either be inhibitory (i.e., becoming negative) or become more excitatory after model fitting is performed. The intrinsic or self-connections are modelled as inhibitory only as they represent a recurrent activity within a region. A more detailed description of spDCM is provided in the Supporting Information. Since the development of spDCM, a number of studies have used this method in order to establish the directed excitation/inhibition between brain areas in the various contexts, such as, recently in relation to emotional intelligence (Bajaj & Killgore, 2021), internet gaming disorder (Dong et al., 2021), or dementia (Benhamou et al., 2020).

### First-Level spDCM Analysis

**Figure 2.**
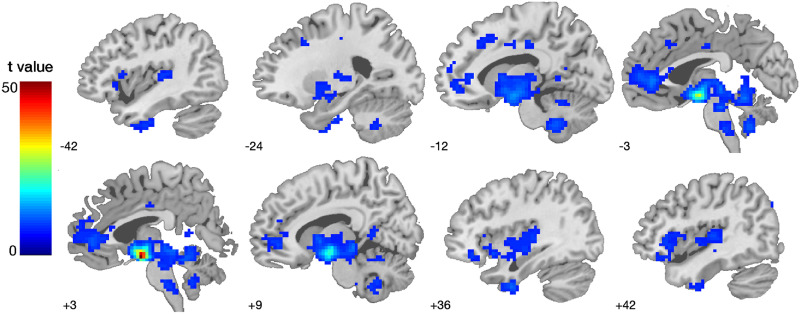
Functional connectivity network of the lateral and medial hypothalamus. Seed-based functional connectivity results by using the bilateral seed mask LH and MH (Figure 1). Results reflect whole-brain one-sample *t* tests at *p* < 0.001 FWE-corrected. For differences in MH and LH functional connectivity, refer to Table 2. The colour bar represents voxel T values.

In order to assess the effective connectivity of the hypothalamus network, regions revealed by the initial functional connectivity analyses of both the MH and LH in conjunction with a minimum voxel size of 20 were used as ROIs for the subsequent spDCM analyses (Figure 2, Table 2). As such, our ROIs were not defined based on previous literature (e.g., Kullmann et al., 2014), but rather in a data-driven fashion. This ROI selection approach is statistically valid (Poldrack, 2007) and has been used previously (e.g., Esménio et al., 2019). At the first-level, a fully connected model was created for each participant and each session. Next, we inverted (i.e., estimated) the DCMs using spectral DCM, which fits the complex cross-spectral density using a parameterised power-law model of endogenous neural fluctuations (Razi et al., 2015). This analysis provides measures of causal interactions between regions, as well as the amplitude and exponent of endogenous neural fluctuations within each region (Razi et al., 2015). Model inversion was based on standard variational Laplace procedures (Friston et al., 2007). This Bayesian inference method uses Free Energy as a proxy for (log) model evidence, while optimising the posterior density under Laplace approximation.

**Table 2.** Peak coordinates of hypothalamus intrinsic functional connectivity networks

Local maxima labelling^a^	Brodmann’s area (BA)	Hemisphere	Peak location (x, y, z)^b^	Cluster size^c^	*T* score
**MH** and **LH**					
Substantia nigra, pars compacta (prSN)		R	3, −3, −15	3,407	50.42
Anterior cingulate cortex, pregenual (pACC)	BA32	L	−3, 42, 3	516	12.03
Inferior temporal gyrus (ITG)		R	36, −3, −45	144	11.51
Middle cingulate & paracingulate gyri (MCC)	BA32	L	−9, 24, 33	123	8.85
Inferior temporal gyrus (ITG)	BA6	L	−42, −6, −42	137	8.66
Inferior frontal gyrus, opercular part	BA44	L	−42, 15, 9	47	8.37
Angular gyrus		R	42, −78, 39	27	7.90
**MH > LH**					
Substantia nigra, pars compacta (prSN)		R	3, −3, −12	92	32.66
Lobule IX of cerebellar hemisphere		L	−6, −36, −60	57	7.48
**LH > MH**					
Red nucleus		R	6, −12, −9	978	23.38
Middle frontal gyrus	BA10	L	−39, 39, 24	334	11.14
Inferior frontal gyrus, opercular part		R	48, 9, 27	61	9.98
Putamen		R	30, 15, 0	85	9.82
Anterior cingulate cortex, supracallosal		R	9, 24, 21	301	9.82
Middle frontal gyrus		R	36, 42, 27	265	9.71
Inferior frontal gyrus, opercular part		L	−54, 9, 24	119	9.64
Inferior parietal gyrus, excluding supramarginal and angular gyri		R	51, −36, 45	322	9.63
Inferior parietal gyrus, excluding supramarginal and angular gyri		L	−45, −45, 42	88	9.47
Lobule VIII of cerebellar hemisphere		L	−33, −57, −60	17	9.45
Cuneus		L	−18, −78, 36	64	9.15
Lobule X of cerebellar hemisphere		L	−9, −30, −39	23	8.80
Superior temporal gyrus	BA38	R	54, 15, −9	42	8.63
Middle cingulate gyrus		R	12, −30, 39	42	8.44
Superior frontal gyrus, dorsolateral		R	30, 3, 57	82	8.41
Putamen		R	36, −12, −9	22	7.95
Superior occipital gyrus		R	21, −72, 36	22	7.71

^a^ Labelling based on AAL3 atlas (Rolls et al., 2020).

^b^ Peak voxel location in MNI space.

^c^ The report of the functional network for the hypothalamus network (MH and LH) was limited to regions sized > 20 voxels.

### Second-Level spDCM Analysis

To characterise how group differences in neural circuitry were modulated by BMI and energy state, hierarchical models over the parameters were specified within a parametric empirical Bayes (PEB) framework for DCM (Friston et al., 2016). The five models we used were based on our hypotheses as follows. Firstly, to investigate the effective connectivity of the MH and LH, two PEB models were estimated for the fasted and sated condition separately. These (intercept) models provide the baseline effective connectivity independent of any behavioural measures. Secondly, we were interested in the group difference between fasted versus sated conditions, and in this PEB analysis, we contrasted the DCMs for the fasted against the sated condition whilst controlling for BMI. Thirdly, we were interested in associating effective connectivity with BMI and used BMI as a main regressor of interest whilst controlling for homeostatic condition. Lastly, we were interested in interaction between group factor (fasted vs. sated) and BMI, and in this PEB analysis, we used the interaction between BMI and the group factor (fasted vs. sated) as main variables of interest.

For each of the presented models, all behavioural regressors were mean centred so that the intercept of each model was interpretable as the mean connectivity. We tested the relationships between all covariates (i.e., age, gender, BMI, subjective hunger reports, blood glucose levels; see Supporting Information) and included blood glucose as a mean-centred covariate, as it correlated with BMI in interaction with the experimental condition. We further controlled for age and gender in every model. Hunger condition was a grouping variable, whereas BMI was a continuous variable. BMI was treated as continuous, as opposed to a grouping variable, as our sample contained overweight (BMI = 25–30 kg/m^2^) and obese (BMI > 30 kg/m^2^) individuals.

Bayesian model reduction was used to test all combinations of parameters (i.e., reduced models) within each parent PEB model (assuming that a different combination of connections could exist; Friston et al., 2016) and ‘pruning’ redundant model parameters. Parameters of the set of best-fit pruned models (in the last Occam’s window) were averaged and weighted by their evidence (i.e., Bayesian model averaging) to generate final estimates of connection parameters. To identify important effects (i.e., changes in directed connectivity), we compared models, using log Bayesian model evidence to ensure the optimal balance between model complexity and accuracy, with and without each effect, and calculated the posterior probability for each model as a softmax function of the log Bayes factor. We treat effects (i.e., connection strengths and their changes) with a strong posterior probability > 0.99 (equivalent of very strong evidence in classical inference) as significant for reporting purposes. This posterior probability indicates very strong evidence for effects in Bayesian statistics (Kass & Raftery, 1995).

Finally, in order to determine the predictive validity (e.g., whether BMI can be predicted from the final, reduced spDCM’s individual connections), leave-one-out cross-validation was performed within the PEB framework (Zeidman et al., 2019). This procedure fits the PEB model in all but one participant and predicts the covariate of interest (e.g., homeostatic state) for the left-out participant. This is repeated with each participant to assess the averaged prediction accuracy for each model.

## RESULTS

We first provide the overview of the seed-based functional connectivity analysis, which was conducted for the derivation of the hypothalamic network. Secondly, we describe the causal dynamics within this derived hypothalamic network.

### Seed-Based Functional Connectivity Analyses

The seed-based functional connectivity analyses (combined LH and MH seed mask, Figure 1) revealed a hypothalamic network comprising seven regions in the substantia nigra, anterior and middle cingulate, inferior temporal and frontal gyrus, and the angular gyrus (Table 2). These seed-based functional connectivity results subtending the MH and LH functional connectivity were used to define the network for each participant for the subsequent effective connectivity analysis. The differential contrast (MH > LH) revealed a stronger functional connectivity between the MH and substantia nigra, cerebellum, and precuneus. The lateral hypothalamus (LH > MH contrast) showed stronger functional connectivity to the red nucleus, inferior and middle frontal gyrus, putamen, anterior and middle cingulate cortex, putamen and regions of the parietal, occipital lobe, as well as cerebellum, (*p* < 0.001, FWE-corrected; Table 2, Figure 2).

### Spectral Dynamic Causal Modelling Results

The average variance explained across subject-level DCM inversion was very high (Hunger: *M* = 86.78, *SD* = 3.23, range = 80.20–94.90; Satiety: *M* = 86.66, *SD* = 2.58, range = 80.02–91.68), indicating very good model convergence.

### Effective Connectivity of the MH and LH During the Fasted and Sated Condition

**Figure 3.**
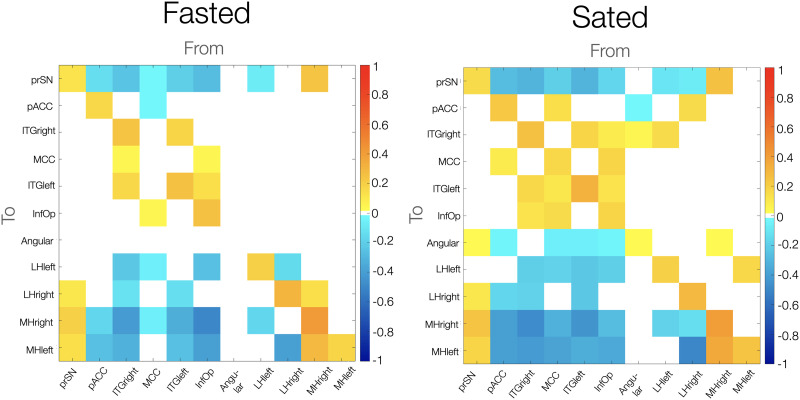
Effective resting-state connectivity of the hypothalamic network across during the fasted versus sated condition. Colour bar indicates effect sizes in hertz. Posterior probability of >.99 (very strong evidence). prSN = substantia nigra pars compacta; MH = medial hypothalamus; LH = lateral hypothalamus; pACC = anterior cingulate cortex pregenual; MCC = middle cingulate and paracingulate gyri; ITG = inferior temporal gyrus; InfOp = inferior frontal gyrus, opercular part.

Figure 3 illustrates the effective connectivity results of the LH and MH during the fasted and sated condition separately. Across the fasted and sated condition, there was inhibition from anterior and mid-cingulate, frontal, temporal and parietal cortex to the LH and MH. There was no evidence for connections going from the LH or MH to these cortical regions in the fasted condition. During the sated condition, there was excitation from the right MH to the angular gyrus, and from the right LH to the pACC.

### Effective Connectivity of the MH and LH in Fasted Versus Sated States

Fasting, compared to satiety, was associated with a decreased excitatory influence from the substantia nigra to the left MH (0.01 Hz, 95% CI [−0.03, 0.004]). The left MH in turn showed an increased excitation to the left LH (0.06 Hz, 95% CI [−0.002, 0.11]) and to the right MH (0.06 Hz, 95% CI [0.007, 0.12]). We further found an increased inhibition from the pregenual anterior cingulate cortex onto the bilateral MH (right hemisphere: 0.14 Hz, 95% CI [0.08, 0.20]; left hemisphere: 0.07 Hz, 95% CI [0.004, 0.14]) and a decreased inhibition from the middle cingulate onto the right MH (−0.01 Hz, 95% CI [−0.03, −0.01]). The left LH in turn received less inhibition from the angular gyrus (−0.01 Hz, 95% CI [−0.02, 0.01]) and exerted stronger inhibition on the substantia nigra (0.01 Hz, 95% CI [−0.007, 0.02]).

**Figure 4.**
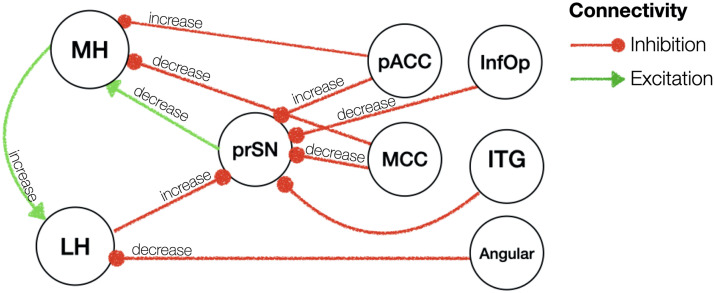
Effective connectivity of fasted versus sated state. Red/green arrows indicate inhibitory/excitatory connectivity. All these connections had posterior probability of >0.99 (very strong evidence). Note that hemispheric information is not shown in this figure for illustration purposes. Please refer to text for details on the hemispheric results. prSN = substantia nigra pars compacta; MH = medial hypothalamus; LH = lateral hypothalamus; pACC = anterior cingulate cortex pregenual; MCC = middle cingulate and paracingulate gyri; ITG = inferior temporal gyrus; InfOp = inferior frontal gyrus, opercular part; Angular = Angular gyrus.

Outside the MH and LH, the substantia nigra pars compacta received a large number of inhibitory inputs from the areas of the cingulate, frontal and temporal cortices. Specifically, during the fasted as opposed to sated state, the substantia nigra pars compacta received a greater inhibition from the bilateral inferior temporal gyrus (left: 0.01 Hz, 95% CI [−0.007, 0.02]; right: 0.01 Hz, 95% CI [−0.007, 0.024]) and the pregenual anterior cingulate cortex (0.01 Hz, 95% CI [−0.02, 0.01]) as well as lower inhibition from the middle cingulate cortex (−0.02 Hz, 95% CI [−0.02, 0.006]) and inferior opercular frontal gyrus (−0.01 Hz, 95% CI [−0.02, 0.01]) (results are summarised in Supporting Information Table S1 and illustrated in Figure 4).

### Effective Connectivity Changes of the MH and LH as a Function of BMI

**Figure 5.**
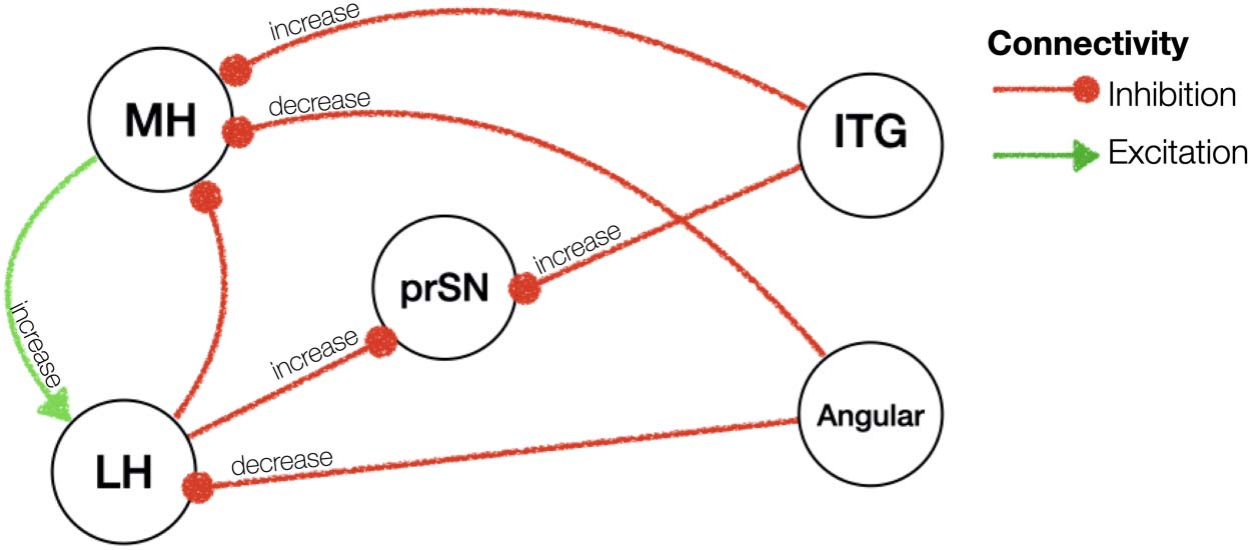
Effective connectivity of BMI. Red/green arrows indicate inhibitory/excitatory connectivity. Note that hemispheric information is not shown in this figure for illustration purposes. Please refer to text for details on the hemispheric results. prSN = substantia nigra pars compacta; MH = medial hypothalamus; LH = lateral hypothalamus; ITG = inferior temporal gyrus; Angular = angular gyrus.

BMI, independent of homeostatic state, was associated with a greater excitatory influence from the left MH to the left LH (0.017 Hz, 95% CI [0.01, 0.03]) and greater inhibition from the right LH to the right MH (0.02 Hz, 95% CI [0.01, 0.02]). We further found a greater inhibition from the inferior temporal gyrus and angular gyrus to the MH (see Table 2 for lateralities and effect sizes). A greater inhibitory influence of the right LH on the substantia nigra (0.007 Hz, 95% CI [0.003, 0.012]) and the right MH (0.02 Hz, 95% CI [0.02, 0.024]) was also evident in individuals with a higher BMI. The substantia nigra pars compacta in turn received a greater inhibition from the bilateral inferior temporal gyrus (left: 0.014 Hz, 95% CI [0.01, 0.02]; right: 0.008 Hz, 95% CI [0.004, 0.01]) (results are summarised in Supporting Information Table S2 and illustrated in Figure 5).

### Effective Connectivity Changes in Fasted Versus Sated States in Interaction With BMI

**Figure 6.**
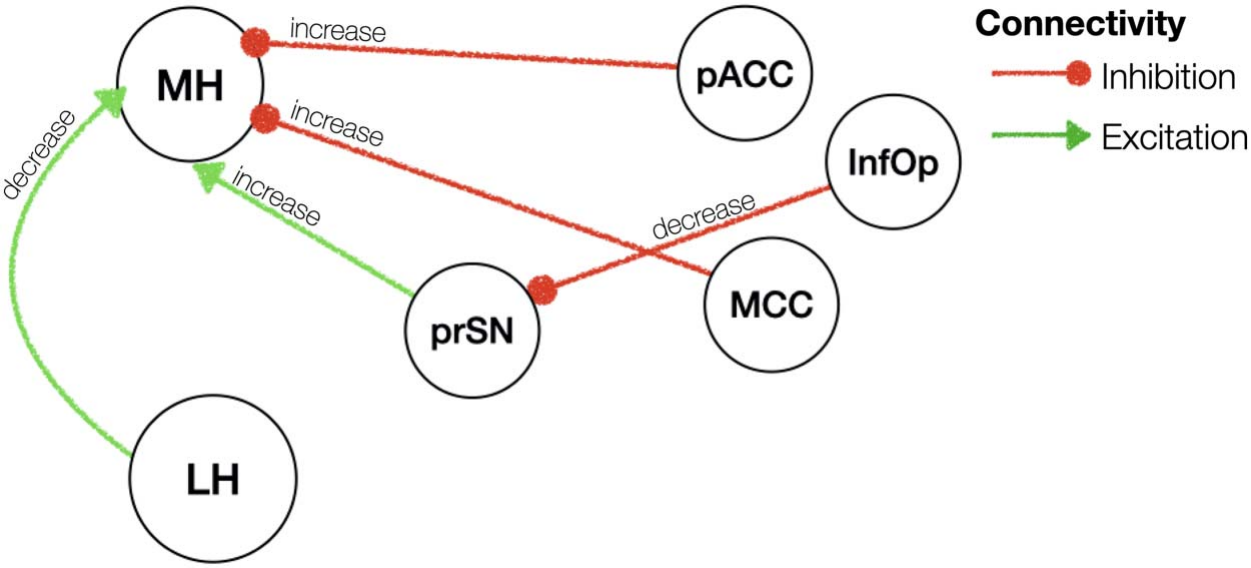
Effective Connectivity of BMI × Homeostatic state interaction effect. Red/green arrows indicate inhibitory/excitatory connectivity. Note that hemispheric information is not shown in this figure for illustration purposes. Please refer to text for details on the hemispheric results. prSN = substantia nigra pars compacta; MH = medial hypothalamus; LH = lateral hypothalamus; pACC = anterior cingulate cortex pregenual; MCC = middle cingulate and paracingulate gyri; ITG = inferior temporal gyrus; InfOp = inferior frontal gyrus, opercular part.

In the final analysis, we investigated how fasted-related connectivity changes were modulated by differences in BMI (Supporting Information Table S3 and Figure 6). During fasting relative to satiety, higher BMI was associated with a higher excitatory influence from the substantia nigra to the left MH (0.006 Hz, 95% CI [0.002, 0.009]). The left MH received an increased excitation from the right MH (0.013 Hz, 95% CI [0.004, 0.02]) as well as a decreased excitation from the left LH (−0.014 Hz, 95% CI [−0.02, −0.005]). The left MH also received higher inhibition from the anterior (0.001 Hz, 95% CI [0, 0.001]) and middle cingulate cortex (0.001 Hz, 95% CI [0, 0.002]). The substantia nigra pars compacta received a greater inhibition from the inferior opercular frontal gyrus (0.006 Hz, 95% CI [−0.01, −0.002]) (results are summarised in Supporting Information Table S3 and illustrated in Figure 6).

## DISCUSSION

This is the first study to reveal how the LH and MH causally interact with other neural regions and how their dynamics change with weight and energy state in humans. Adopting state of the art spectral dynamic causal modelling of resting-state fMRI data (Friston et al., 2014; Park et al., 2018; Razi et al., 2015), our results show two core networks interacting: (a) subcortical bidirectional connections between the LH, MH, and the prSN, and (b) cortical top-down inhibition from frontal, cingulate, and temporal onto the subcortical network. The prSN seems to represent a central hub interconnecting the subcortical and cortical neural systems. During the fasted compared to the sated state, regardless of weight status, we found increased inhibition between the right LH and prSN as well as decreased excitation between the prSN and left MH, whereas the prSN received top-down inhibition from across the cortex, which may represent an adaptive motivational drive to seek food while hungry, in fitting with animal studies (Cassidy & Tong, 2017; Nieh et al., 2016; Rossi et al., 2019; Rossi & Stuber, 2018). However, individuals with excess weight revealed a similar hypothalamic network communication irrespective of being in a fasted or sated state. Further, when taking into consideration excess weight, they showed a reverse communication pattern of decreased substantia nigra-MH inhibition during the fasted state. The neural network communications involved in the regular processes of food seeking after fasting may therefore be disrupted in individuals with excess weight, providing a compelling hypothesis for food overconsumption beyond metabolic needs. We previously used spDCM to investigate how the food choice network, which includes the hypothalamus relates to BMI and homeostasis. Here, we found that BMI and homeostatic state modulated the hypothalamus connections to subcortical and cortical areas (Voigt et al., 2021). However, this work did not distinguish between the LH and MH, which are known to have significant different functionalities (Anand & Brobeck, 1951; Brobeck et al., 1943; Elmquist et al., 1999; Hetherington & Ranson, 1983), and did not specifically focus on the hypothalamic network as established in this work, but rather on a food choice network (Voigt et al., 2021).

Our results from whole-brain functional connectivity analyses largely reflect those reported in earlier neuroimaging studies (Kullmann et al., 2014; Martín-Pérez et al., 2019; Zhang et al., 2018). Both resting-state activity of LH and the MH was correlated with resting-state activity of prSN, middle and anterior cingulate cortex, inferior frontal and temporal gyrus as well as angular gyrus. Consistent with animal and human research, the LH was more strongly connected than the MH across the entire brain, ranging from subcortical areas (e.g., red nucleus, putamen) to all neocortical areas. This finding strengthens the established role of the LH as a central interface integrating diverse central and peripheral signals through a complex large-scale neural network that may coordinate adaptive behavioural responses related to motivation and controlled feeding behaviour (Bonnavion et al., 2016; Petrovich, 2018). The MH in turn was more strongly connected to the prSN, cerebellum, and precuneus. These and previous findings (Kullmann et al., 2014; Martín-Pérez et al., 2019; Zhang et al., 2018) highlight the potential of a dual hypothalamic functionality resulting from distinct LH and MH neural networks. However, these characterisations have been limited as the directionality and valence of the interactions within these networks have remained unknown. Here, we have extended the characterisation of these networks by means of spDCM, investigating the directed communication of the LH and MH networks and their changes as a function of both BMI and energy state.

Irrespective of BMI and energy state, the prSN emerges as a key area connecting the hypothalamus with neocortical regions. The prSN processes autonomic, gut-induced rewards regulating motivational and emotional states (e.g., Gutierrez et al., 2020; Han et al., 2018). Hormones implicated in regulating the homeostatic system also impinge directly on dopamine neurons in the prSN (Palmiter, 2007). Anatomically and functionally the prSN is highly interconnected with the ventral tegmental area (VTA), and both regions contribute to motivation and reward processing (Ilango et al., 2014; Kwon & Jang, 2014). In hungry mice, inhibitory inputs from the LH to the VTA inhibited dopamine release, resulting in increased motivation to seek and approach food (Nieh et al., 2016). In this study we found an increased inhibitory influence from the LH to the prSN when participants were hungry. Given the strong prSN-VTA interconnectivity and interchangeable functionality (Ilango et al., 2014; Kwon & Jang, 2014), it is reasonable to assume that the increased inhibition from the LH to the prSN in humans might mirror a necessary survival mechanism to increase appetitive motivation to prevent starvation. Notably, in individuals with higher BMI, regardless of their energy state, this inhibition from LH to prSN persisted. This failure to ‘shut off’ the inhibitory signalling might reflect an underlying neural trigger for increased motivation for food regardless of homeostatic state (Berthoud, 2004, 2012; Berthoud et al., 2017; Cassidy & Tong, 2017).

During the fasted compared to the sated state, we also found a decreased excitation from the prSN to the MH. The MH contains a diverse array of nuclei and circuits, including the anorectic melanocortin system that reduces food consumption, as well as increasing energy expenditure (Kühnen et al., 2019). Thus, the reduced excitation during fasting compared to satiety may reflect reduced activation of this anorectic pathway. However, it is also important to note that the MH contains strong drivers of appetite and motivation. Agouti-related peptide neurons in the hypothalamic arcuate nucleus drive food intake and motivation (Andermann & Lowell, 2017). In individuals with excess weight, this excitatory connectivity between prSN and MH increased during the fasted compared to the sated state. This might contribute to increased food seeking and consumption in response to energy deprivation among individuals with overweight/obesity (Kühnen et al., 2019). Clearly, future research in humans is required to examine the activity of specific hypothalamic nuclei within the MH region in relation to food seeking and obesity; however, this is currently beyond the technical capability of MRI.

In addition to the communication between MH and LH with the prSN, we also found intrahypothalamic connectivity between the LH and MH across weight and homeostatic state. The dynamic from the ventral MH to the LH has been previously observed in animals (Canteras et al., 1994; Horst & Luiten, 1986; Luiten et al., 1980); however, its functional significance remains untested and further studies in humans are needed to elucidate the functionality of intrahypothalamic connectivity patterns.

During the fasted as opposed to the sated state, we found an increased top-down inhibition from the pregenual anterior cingulate cortex to the MH, yet a decreased top-down inhibition from the middle cingulate gyrus to the MH and prSN. While it is not possible to definitively disentangle the role of these network dynamics in the current context, previous neuroimaging studies that have dissociated the functions of the pregenual anterior cingulate cortex and the middle cingulate gyrus provide for some conjecture (Stevens et al., 2011). In particular, spontaneous activity in the pregenual anterior cingulate cortex is associated with affective processing and anticorrelated with activity in sensorimotor areas. In contrast, activity in the middle cingulate gyrus is temporally coupled with activity in sensorimotor areas, and functionally connected with areas involved in cognitive control. The causal dynamics we report herein between the middle cingulate gyrus, pregenual anterior cingulate cortex, and MH might therefore suggest interactions between homeostatic inputs and affective, sensorimotor, and cognitive networks dynamics. Note that the increased inhibition from the pregenual anterior cingulate cortex to the MH was not related to BMI. However, increased inhibition from the middle cingulate gyrus to the MH was exacerbated in participants with higher BMIs. Future studies are needed to clarify if this alteration is associated with core symptoms of obesity.

In addition to the cingulate cortex, the angular gyrus had a decreased impact on the LH, when individuals were fasted or in individuals with excess weight. Furthermore, all cortical areas inhibited either the prSN or hypothalamus irrespective of weight and homeostatic state. Whereas the cingulate cortex is a hub for sensory, motivational and cognitive information, the prefrontal and parietal cortex are more predominantly associated with executive control (Seeley et al., 2007). In participants with excess weight, a differential pattern within the executive control network has been observed in fMRI activation studies using food stimuli (Franssen et al., 2020). Recently, it has also been shown that obesity is related to prominent functional connectivity alterations mainly in prefrontal regions during resting-state as well as in response to food stimuli (García-García et al., 2013; Kullmann et al., 2012). Thus, our resting-state findings might further add to the possibility of disrupted communication between the executive control network and regions regulating metabolic needs in individuals with excess weight.

In the light of the proposed mechanisms here, we note, however, that the relationship between resting-state effective connectivity and its cognitive correlates remains elusive. The interindividual variations in effective connectivity do not necessarily overlap with the interindividual variations in effective connectivity during task performance (Fox & Raichle, 2007; Jung et al., 2018). At this stage, only one study has revealed that resting-state effective connectivity might facilitate task performance but may not reflect task-based network dynamics (Jung et al., 2018). Future studies are needed to address whether the resting-state dynamics revealed in our study are also engaged during task performance and how potential deviations might translate to differences in behaviour or clinical phenotypes.

Food intake is orchestrated by a large-scale network of subcortical and cortical areas on distinct higher and lower order cognitive functions (de Araujo et al., 2020). Both hemispheres need cross talk to support functions such as food seeking and balanced food intake (Voigt et al., 2021). Our results support this view by revealing that there is no specific hemisphere favoured and regions occur equally distributed in both, in the right and left hemisphere of the brain. Previous studies included the bilateral MH/LH, but did not reveal whether lateralisation might be functionally significant (Kullmann et al., 2014; Zhang et al., 2018), and reviews or opinion papers on that matter did not discuss laterality (Petrovich, 2018). Although there has been some animal research discussed that the hypothalamus might have functional lateralisation (Kiss et al., 2020), more research is needed to systematically test this claim in humans and in relation to BMI and homeostasis. As such, in this discussion, we do not distinguish between the left and right hemisphere and discussed each region functionality and bilaterally.

In conclusion, our study provides new insights into how hunger and satiety states affect hypothalamic circuit dynamics, which involve a distributed network of midbrain and cortical areas with a key role of the substantia nigra pars compacta. We also identified unique aspects of network organisation associated with obesity, which involve the reciprocal connections between the lateral and MH, and the input from the substantia nigra to the MH.

## ACKNOWLEDGMENTS

The authors thank Richard McIntyre, Naomi Kakoschke, Amelia Romei, Tori Gaunson, and Tiffany Falcone for help with MRI data acquisition.

## SUPPORTING INFORMATION

Supporting information for this article is available at https://doi.org/10.1162/netn_a_00266. Data for main spectral dynamic causal modelling are available at GitHub: https://github.com/KatharinaVoigt1/spDCM_Hypo.git (Voigt, 2021).

## AUTHOR CONTRIBUTIONS

Katharina Voigt: Conceptualization; Formal analysis; Project administration; Visualization; Writing – original draft; Writing – review & editing. Zane B. Andrews: Conceptualization; Funding acquisition; Writing – review & editing. Ian H. Harding: Conceptualization; Formal analysis; Funding acquisition; Writing – review & editing. Adeel Razi: Formal analysis; Methodology; Writing – review & editing. Antonio Verdejo-Garcia: Conceptualization; Funding acquisition; Writing – review & editing.

## FUNDING INFORMATION

Antonio Verdejo-García, Zane Andrews, and Ian Harding, NHMRC Grant, Award ID: GNT1140197.

## Supplementary Material



## TECHNICAL TERM

Spectral dynamic causal modelling (spDCM): spDCM is a method for analyzing resting-state functional magnetic resonance imaging (fMRI) data that provides information about directionality (inhibition vs. excitation) of connectivity between brain areas.

## Supporting Information

https://github.com/katharinavoigt1/spdcm_hypo.git
