# Supplementary material for: Hypothalamic effective connectivity at rest is associated with body weight and energy homeostasis
Source: Netw Neurosci. 2022 Oct 1;6(4):1316–33. doi: 10.1162/netn_a_00266 (PMC11117096; doi:10.1162/netn_a_00266)
Supplement: Supplementary file 1 [file netn-6-4-1316-s001.pdf]

Voigt, K., Andrews, Z. B., Harding, I. H., Razi, A. & Verdejo-Garcia, A. (2022). Supporting information for "Hypothalamic effective connectivity at rest is associated with body weight and energy homeostasis." *Network Neuroscience*. Advance publication. https://doi.org/10.1162/netn_a_00266

Supplementary Materials

Supplementary Methods

Spectral Dynamic Causal Modelling

Dynamic causal modelling (DCM) is Bayesian framework that infers the directed (causal) connectivity among the neuronal systems – referred to as effective connectivity. We recently proposed a new DCM for resting state fMRI – based upon a deterministic model that generates predicted cross spectra – referred to as spectral DCM (Friston et al., 2014). In order to model resting state activity – in the absence of external stimuli – we will have to add a stochastic component, i.e. neural fluctuations, to the classical DCM based on ordinary differential equations. Mathematically, we can express the formulation of the stochastic generative model using a set of two equations. First is the neuronal state equation, namely

$$\dot{x}(t) = f(x(t), u(t), \theta) + v(t), \qquad \text{(S1)}$$

and second is the observation equation, which is a static nonlinear mapping from the hidden physiological states in (1) to the observed BOLD activity and is written as:

$$y(t) = h(x(t), \varphi) + e(t), \qquad \text{(S2)}$$

where $\dot{x}$(t) is the rate of change of the neuronal states $x(t)$, θ are unknown parameters (i.e., the effective connectivity) and $v(t)$ (resp. $e(t)$) is the stochastic process – called the state noise (resp. the measurement or observation noise) – modelling the random neuronal fluctuations that drive the resting state activity. In the observation equations, φ are the unknown parameters of the (haemodynamic) observation function and $u(t)$ represents any exogenous (or experimental) inputs that drive the hidden states – that are usually absent in resting state designs (Friston et al., 2014). Spectral DCM furnishes a constrained inversion of

the stochastic model by parameterising the neuronal fluctuations $v(t)$. Spectral DCM simplifies the generative model by replacing the original timeseries with their second-order statistics (i.e., cross spectra). This means, instead of estimating time varying hidden states, we are estimating their covariance which is time invariant. Then we simply need to estimate the covariance of the random fluctuations; where a scale free (power law) form for the state noise (resp. observation noise) is used – motivated from previous work on neuronal activity (Beggs & Plenz, 2003) – as follows:

$$g_v(\omega, \theta) = \alpha_v \omega^{-\beta_v}$$

$$g_e(\omega, \theta) = \alpha_e \omega^{-\beta_e} \tag{S3}$$

Here, $\{\alpha, \beta\} \subset \theta$ are the parameters controlling the amplitudes and exponents of the spectral density of the neural fluctuations. The parameterisation of endogenous fluctuations means that the states are no longer probabilistic; hence the inversion scheme is significantly simpler, requiring estimation of only the parameters (and hyperparameters) of the model.

We used standard Bayesian model inversion to infer the parameters of the model in (1), (2) and (3), from the observed signal $y(t)$. The description of the Bayesian model inversion procedures based on variational Laplace can be found elsewhere for the interested readers (Friston et al., 2007).

Supplementary Analyses

Relationship between BMI and Subjective Hunger Reports.

There was no significant relationship between BMI and reported subjective hunger during the fasted (M = 4.42, SD = 1.44) and sated condition (M = 3.22, SD = 1.48) (Main effect of subjective hunger on BMI: β = -0.06, *SE* = 0.18, *p* = .76; Interaction effect between subjective

hunger and homeostatic condition on BMI: $\beta = 0.17$, SE = 0.25, p = .51) (Figure S1). When excluded an outlier based on BMI (ID_1 = 55.56 kg/m2), this relationship remained.

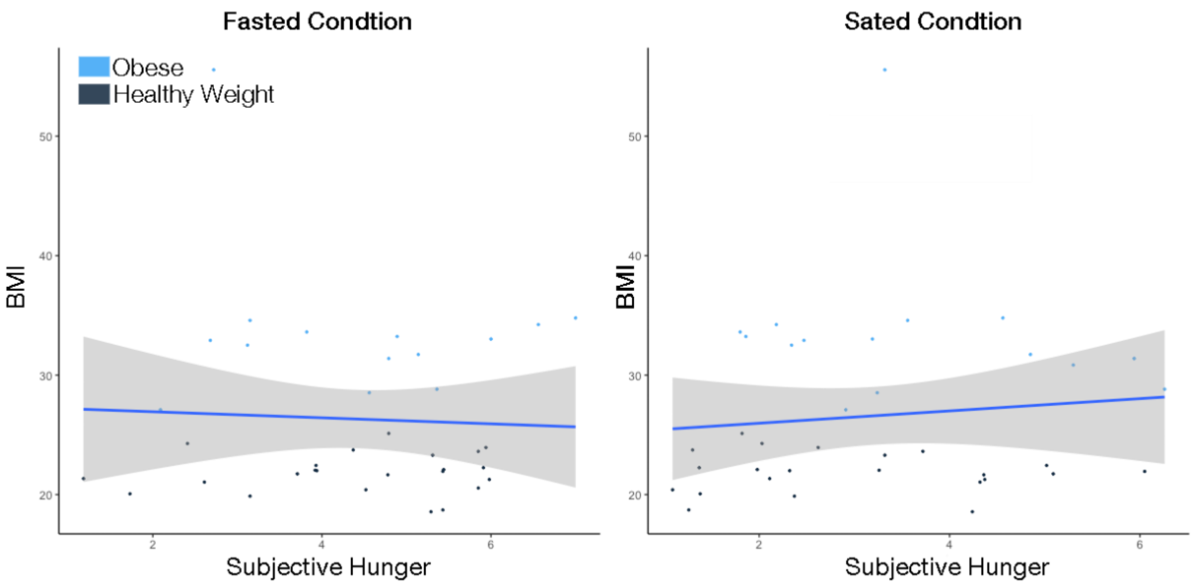

Figure S1. Relationship between reported hunger and BMI across the fasted and sated condition. Grey area indicates 95% confidence interval of linear model's predictions.

Relationship between BMI and Fasting Blood Glucose Levels.

There was a significant positive relationship between BMI and fasting blood glucose levels: with each increase in BMI by 1 kg/m^2, blood glucose levels increased by 0.31 mg/dL ($\beta = 0.31$, SE = 0.15, p = .04) (Figure S2, left panel). However, when excluding outliers in BMI (ID_1 = 55.56 kg/m2) and blood glucose levels (ID_18 = 6.4 mg/dL; ID_23 = 8.4 mg/dL), this relationship did not persist (Figure S2, right panel), ($\beta = 0.27$, SE = 0.28, p = .33). In order to avoid potentially extracting meaningful network information by excluding these participants, we instead controlled for fasting blood glucose levels when investigating the dynamics of the hypothalamic neural network model in relation with BMI (i.e. Model 2, Model 3, and Model

4).

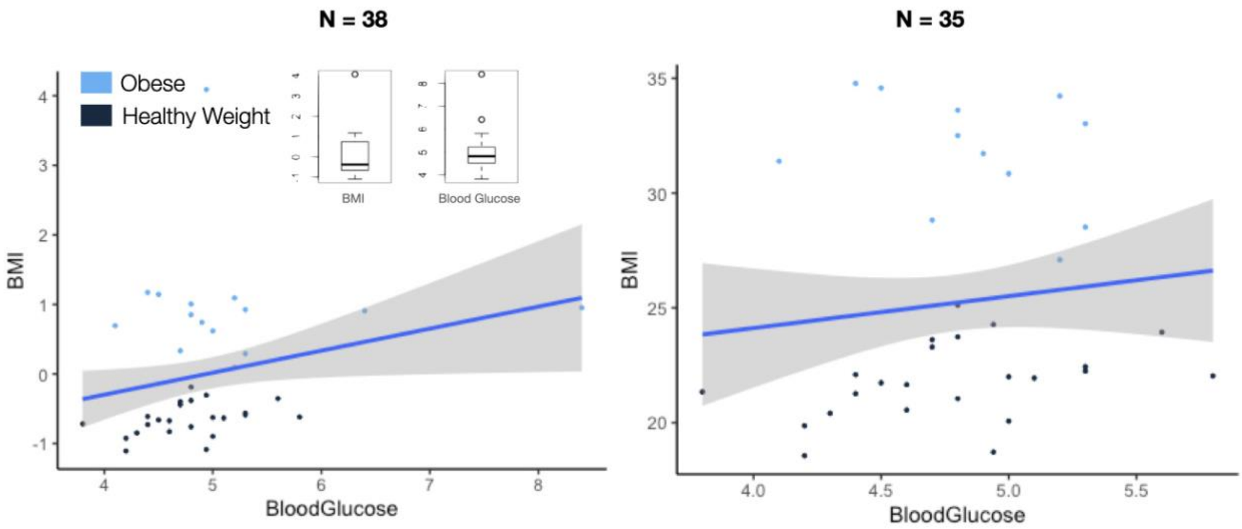

Figure S2. Relationship between BMI and fasting blood glucose levels for all participants (N = 38, left panel) and for when outliers were excluded (N = 35, right panel). Grey area indicates 95% confidence interval of linear model's predictions.

Supplementary Results

Table S1. Modulation of the Hypothalamic Network by Homeostatic State (Fasted vs. Sated)

Parity	Connection	Fasted vs. Satiety Effect (↑/↓)	Effect size in Hz [95% CI]
Excitation	prSN → MH_{left}	↓	0.01 [-0.03, 0.004]
	MH_{left} → MH_{right}	↑	0.06 [0.007, 0.12]

	MH_{right} → LH_{left}	↑	0.06 [-0.002, 0.11]
Inhibition	pACC → MH_{right}	↑	0.14 [0.08, 0.20]
	pACC → MH_{left}	↑	0.07 [0.004, 0.14]
	MCC → MH_{right}	↓	0.02 [-0.03, -0.01]
	Angular → LH_{left}	↓	0.004 [-0.02, 0.01]
	LH_{right} → prSN	↑	0.01 [-0.007, 0.02]
	pACC → prSN	↑	0.07 [0.045, 0.1]
	ITG_{right} → prSN	↑	0.01 [-0.007, 0.024]
	MCC → prSN	↓	0.01 [-0.02, 0.006]
	ITG_{left} → prSN	↑	0.01[-0.01, 0.02]
	InfOp → prSN	↓	0.01 [-0.02, 0.01]

Note: CI, confidence interval; prSN, substantia nigra pars compacta; MH, medial hypothalamus; LH, lateral hypothalamus; pACC, anterior cingulate cortex pregenual; MCC,

middle cingulate and paracingulate gyri; ITG, inferior temporal gyrus; InfOp, inferior frontal gyrus, opercular part; ↑, increases in connectivity; ↓, decreases in connectivity.

Table S2. Differences in the Hypothalamic Network associated with BMI

Parity	Connection	BMI effect	Effect size in Hz [95% CI]
Excitation	$MH_{left} \rightarrow LH_{left}$	↑	0.017 [0.01, 0.03]
Inhibition	$ITG_{right} \rightarrow MH_{right}$	↑	0.02 [0.01, 0.03]
	$ITG_{right} \rightarrow MH_{left}$	↑	0.002 [0, 0.003]
	$ITG_{left} \rightarrow MH_{right}$	↑	0.02 [0.01, 0.03]
	$ITG_{left} \rightarrow MH_{left}$	↑	0.02 [0.01, 0.03]
	Angular → MH_{left}	↓	0.01 [-0.01, -0.003]
	Angular → LH_{left}	↓	0.004 [-0.008, -0.001]
	$LH_{right} \rightarrow MH_{right}$	↑	0.02 [0.01, 0.024]

	LH_{right} → prSN	↑	0.007 [0.003, 0.012]
	ITG $_{right}$→ prSN	↑	0.008 [0.004, 0.01]
	ITG $_{left}$ → prSN	↑	0.014 [0.01, 0.02]

Note: CI, confidence interval; prSN, substantia nigra pars compacta; MH, medial hypothalamus; LH, lateral hypothalamus; pACC, anterior cingulate cortex pregenual; MCC, middle cingulate and paracingulate gyri; ITG, inferior temporal gyrus; InfOp, inferior frontal gyrus, opercular part; ↑, increases in connectivity; ↓, decreases in connectivity.

Table S3. Hypothalamic network associated with BMI x Homeostatic State interaction

Parity	Connection	BMI x Homeostatic state interaction effect	Effect size in Hz [95% CI]
Excitation	prSN → MH_{left}	↑	0.006 [0.002, 0.009]
	LH_{left} → MH_{left}	↓	0.014 [-0.02, -0.005]
	MH_{right}→MH_{left}	↑	0.013 [0.004, 0.02]

Inhibition	pACC→ MH_{left}	↑	0.001 [0, 0.0014]
	MCC → MH_{left}	↑	0.001 [0, 0.002]
	InfOp → prSN	↑	0.006 [-0.01,-0.002]

Note: CI, confidence interval; prSN, substantia nigra pars compacta; MH, medial hypothalamus; LH, lateral hypothalamus; pACC, anterior cingulate cortex pregenual; MCC, middle cingulate and paracingulate gyri; ITG, inferior temporal gyrus; InfOp, inferior frontal gyrus, opercular part; ↑, increases in connectivity; ↓, decreases in connectivity.

Supplementary References

Friston, K.J., Kahan, J., Biswal, B., Razi A. A DCM for resting state fMRI. *NeuroImage* 2014; 94, 396–407.

Beggs, J.M., Plenz, D. (2003). Neuronal Avalanches in Neocortical Circuits. *J Neurosci*, 23, 11167.

Friston, K., Mattout, J., Trujillo-Barreto, N., Ashburner, J., Penny, W. (2007). Variational free energy and the Laplace approximation. *NeuroImage*, 34: 220–234.
